# Imaging the response to deep brain stimulation in rodent using functional ultrasound

**DOI:** 10.1088/1361-6560/abdee5

**Published:** 2021-02-24

**Authors:** Rohit Nayak, Jeyeon Lee, Siobhan Chantigian, Mostafa Fatemi, Su-Youne Chang, Azra Alizad

**Affiliations:** 1 Department of Radiology, Mayo Clinic College of Medicine and Science, Rochester, Minnesota 55902, United States; 2 Department of Neurologic Surgery, Mayo Clinic College of Medicine and Science, Rochester, Minnesota 55902, United States; 3 Department of Physiology and Biomedical Engineering, Mayo Clinic College of Medicine and Science, Rochester, Minnesota 55902, United States

**Keywords:** deep brain stimulation, functional ultrasound, thalamus stimulation, microvasculature imaging, cerebral hemodynamics

## Abstract

In this study, we explored the feasibility of using functional ultrasound (fUS) imaging to visualize cerebral activation associated with thalamic deep brain stimulation (DBS), in rodents. The ventrolateral (VL) thalamus was stimulated using electrical pulses of low and high frequencies of 10 and 100 Hz, respectively, and multiple voltages (1–7 V) and pulse widths (50–1500 *μ*s). The fUS imaging demonstrated DBS-evoked activation of cerebral cortex based on changes of cerebral blood volume, specifically at the primary motor cortex (PMC). Low frequency stimulation (LFS) demonstrated significantly higher PMC activation compared to higher frequency stimulation (HFS), at intensities (5–7 V). Whereas, at lower intensities (1–3 V), only HFS demonstrated visible PMC activation. Further, LFS-evoked cerebral activation was was primarily located at the PMC. Our data presents the functionality and feasibility of fUS imaging as an investigational tool to identify brain areas associated with DBS. This preliminary study is an important stepping stone towards conducting real-time functional ultrasound imaging of DBS in awake and behaving animal models, which is of significant interest to the community for studying motor-related disorders.

## 1. Introduction

Deep brain stimulation (DBS) has demonstrated substantial efficacy in treatment of movement-related disorders (essential tremor, Parkinson’s disease and dystonia) and psychiatric conditions (obsessive-compulsive disorder) (Grill *et al* 2004, McIntyre *et al* 2004, Johnson *et al* 2008, Benabid *et al* 2009, Jackson and Zimmermann 2012). Although it has proven to be an effective alternative treatment modality to medication, the understanding of its underlying mechanism is still limited and under active exploration (McIntyre *et al* 2004, Ressler and Mayberg 2007, Johnson *et al* 2008, Pienaar *et al* 2015). Brain stimulation can evoke neurological response both locally or globally across the brain. Comprehensive study of these DBS-evoked activation in the brain using electrophysiology can be limited due to constraints on spatial sampling points.

Several researchers have used functional magnetic resonance imaging (fMRI) to map neurological activation and functional connections on a whole-brain scale, in response to DBS at various targets (Shyu *et al* 2004, Arantes *et al* 2006, Yang *et al* 2013, Chao *et al* 2014, Lai *et al* 2014, Younce *et al* 2014, Paek *et al* 2015, Van Den Berge *et al* 2017). However, a major limitation associated with fMRI is the need for immobilization of the animal, which leads to use of anesthesia and muscle relaxants that: (1) may have confounding impact on the stimulus evoked neurological response, (2) excludes neuroimaging of behaving animals, especially in motor-ability related neurological diseases.

Recent developments in high frame-rate ultrasound imaging and advanced spatiotemporal clutter filtering have significantly improved sensitivity of blood flow imaging, without the use of contrast agents (Macé *et al* 2011, Osmanski *et al* 2014, Demené *et al* 2015, Sieu *et al* 2015, Demene *et al* 2017, Tiran *et al* 2017, Deffieux *et al* 2018, Demené *et al* 2019, Rabut *et al* 2019, Sauvage *et al* 2019). Improved blood flow signal estimation allows detection of subtle blood variations in small arterioles (down to 1 mm s^−1^) that are associated with neuronal activity (Mace *et al* 2013, Tanter and Fink 2014, Deffieux *et al* 2018). In fUS imaging, the activation signal is directly proportional to the concentration of dynamic red blood cells in the local volume (Mace *et al* 2013), which makes it a very valuable tool for understanding brain functioning and connectivity with respect to different kinds of stimulus, in both resting, awake and behaving animal models (Aydin *et al* 2020). This aspect of fUS imaging is an important factor in preclincal studies, for neuroimaging of animal models of movement related disorders because motor response captured in animal behavior can be directly correlated with the neurological signal in the primary motor cortex (PMC). Several researchers have used fUS neuroimaging to study visual (Gesnik *et al* 2017, Macé *et al* 2018), somatosensory (Urban *et al* 2014, 2015), olfactory (Osmanski *et al* 2014, Rungta *et al* 2017, 2018) evoked responses in anesthetized (Macé *et al* 2011, Mace *et al* 2013) and awake rodents (Urban *et al* 2015, Tiran *et al* 2017), which has also been applied to humans (Demene *et al* 2017), pigeons (Rau *et al* 2018), ferrets (Bimbard *et al* 2018) and monkeys (Dizeux *et al* 2019). In this study, we will test the feasibilityof using fUS imaging to characterize the brain activity induced by DBS at VL thalamus in rodents. The hypothesis of this study is that functional ultrasound imaging can visualize neuronal activation associated with thalamic DBS with high spatial and temporal resolution. We tested this hypothesis on rodents using a programmable ultrasound scanner.

## 2. Methods

### 2.1. Animal handling and surgery

A total of 4 rats were considered in this study for DBS-fUS imaging experiments. All experimental procedures were approved by the Mayo Clinic Institutional Animal Care and Use Committee for Experimental Animals. Adult Sprague Dawley rats weighing 250–400 g were used in this study. Rats were anesthetized by a combination of ketamine (40 mg kg^−1^) and dexmedetomine (0.15 mg kg^−1^). The anesthesia depth was checked with hind paw and tail pinch. Once the animal was fully anesthetized, it was transferred to a stereotaxic frame (David Kopf Instruments, USA). Supplementary doses of ketamine/dexmedetomidine (12.5 mg kg^−1^ and 0.05 mg kg^−1^, respectively) were injected every hour until the experiment was completed. After a midline incision, a craniotomy was performed. The cranial window was 5 mm wide along the medial-lateral (ML) direction (coordinates from +4 to −1 mm relative to bregma) and 12 mm long along the anterior–posterior (AP) direction (coordinates from +4 to −8 mm relative to bregma). Before the craniotomy, the midline was marked on the skin to align the center of the transducer (figures 2(a), (b)). All procedures were accomplished by leaving the underlying dura mater intact to prevent cerebral damage. To apply electrical stimulation to the VL thalamus, a twisted bipolar electrode (stainless steel, Plastics One) was unilaterally implanted to the VL thalamus (AP: −2.4 mm, ML: 1.9 mm and DV: 5.8 mm from surface) (figure 2(a)). In order to avoid physical interference with the ultrasound transducer, the electrodes were formed into a right-angle prior to insertion and anchored on the edge of the bone flap with dental acrylic cement (figure 2(a)). After completing the experiments, we performed histology to confirm the position of the electrode tips using 4% paraformaldehyde (Sigma-Aldrich, St. Louis, MO, USA). The fixed brain was thinly sliced (40 *μ*m) and stained with cresyl violet to clarify the sub-areas of the brain (figure 2(c)), and the electrode position was identified based on atlas by Paxinos and Watson (Paxinos and Watson 2006).

### 2.2. Data acquisition

**Figure 1.**
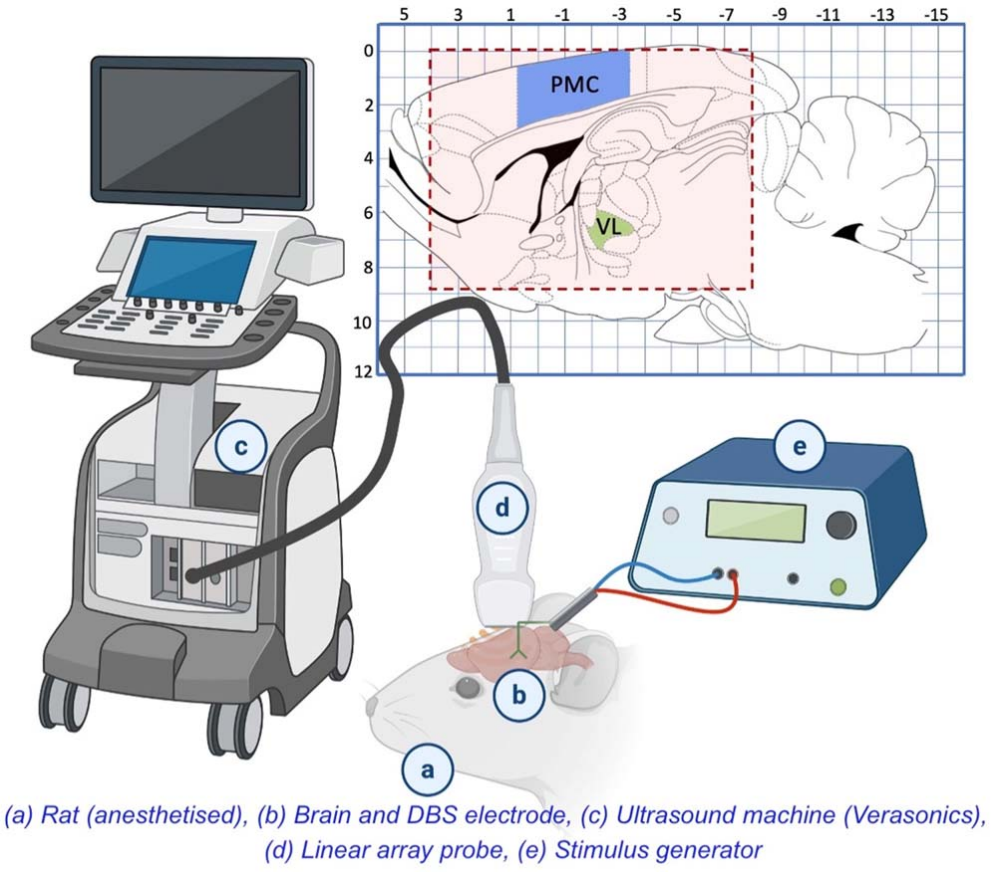
An illustrative depiction of the DBS-fUS experimental setup used in this study. Components (a)–(e) were designed on biorender.com. The schematic of the saggital cross-section of the rat brain corresponds to ML = 1.4 mm (Paxinos and Watson 2006). The regions of interest PMC and VL are indicated in blue and green, respectively. The red ROI outlines the fUS imaging region considered in this study.

**Figure 2.**
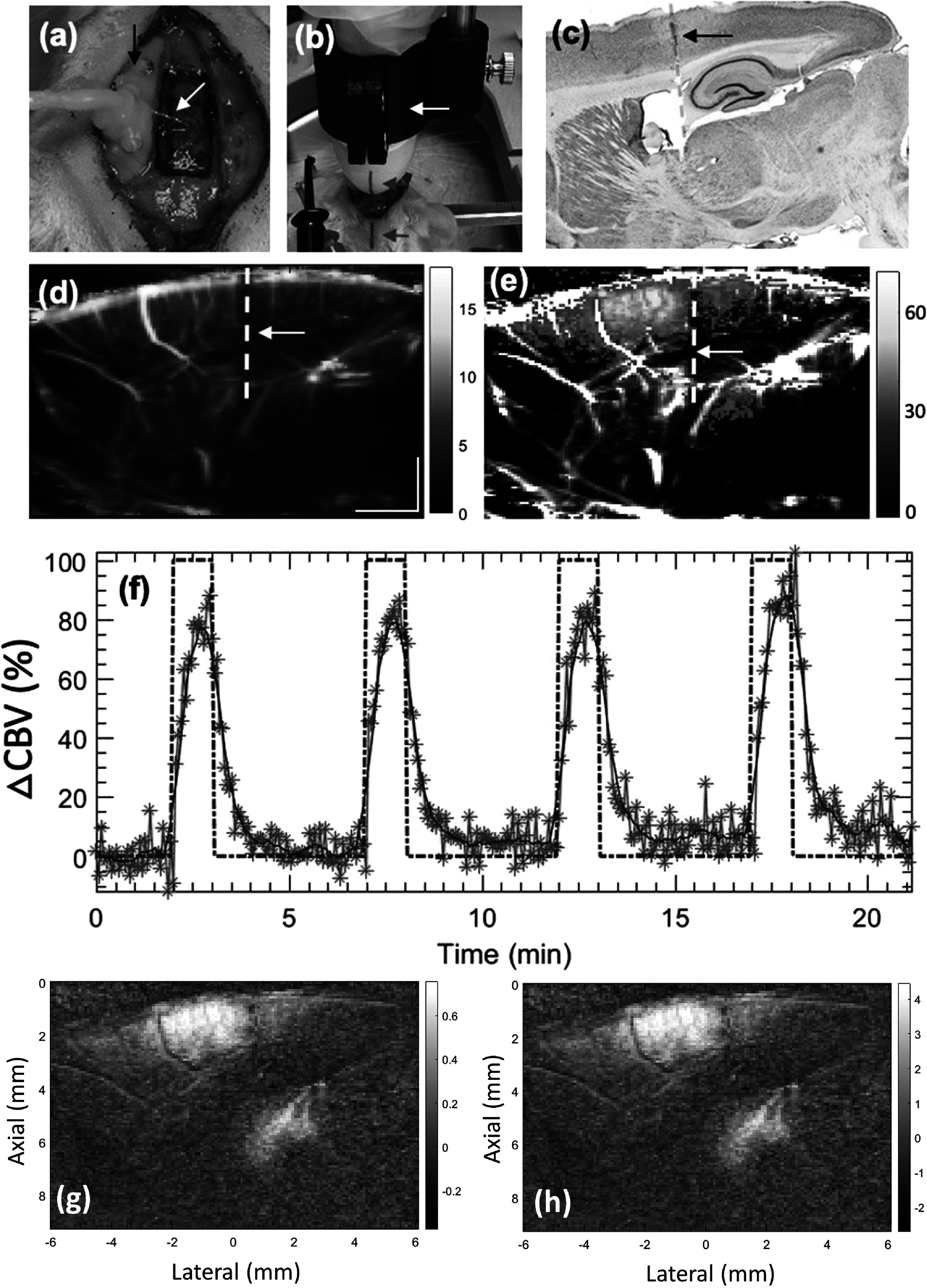
(a) A limited craniotomical window for functional imaging of rat brain, with implanted electrode indicated in white. (b) Experimental setup with a high frequency ultrasound transducer positioned over the craniotomical window, aligned with the mid-line. (c) Histological cross-section of the rat brain stained by cresyl violet to confirm electrode position. (d) A representative ultrasound microDopler image of the rat-brain, imaged at ML = 1.4 mm. The vertical and horizontal scales measure 2 mm. The position of the inserted electrode is indicated in white. (e) displays an instance of fUS image super-imposed on microDoppler image, corresponding to the electrical stimuli at the ventrolateral thalamus. Temporal profile of primary motor cortex activation regulated with on/off phases of deep brain stimulation is displayed in (f), in response to four repeated instances. The blue and red plots indicates on-off DBS phases (activation function), and the corresponding mean fUS signal in the primary motor cortex, respectively. The black plot corresponds to temporally averaged fUS signal indicated in red. (g), (h) depicts the correlation and z-score images, corresponding to (e).

Ultrasound data was acquired using Verasonics Vantage 256 channel ultrasound machine (Verasonics, USA) equipped with a high frequency linear array L22–14 transducer (Vermon, France). For each microDoppler image, 400 IQ frames were acquired at a frame-rate of 1 KHz, every 2 s. The ultrasound data was acquired at a transmit and sampling frequencies of 18.5 and 74 MHz, respectively. Figure 1 displays an illustration of the DBS-fUS experimental setup used in this study. The rat brain schematic depicts the regions of interest and the overall imaging region. The transmit sequence involved compounded plane-wave imaging at five insonification angles, equally spaced between ±14^o^, with a pulse repetition frequency of 5 KHz. Figure 2(b) displays the experimental setup used for fUS imaging of the rat brain in the sagittal plane. The ultrasound probe was positioned directly over the cranial window, centered at the stereotaxic coordinate of ML: 1.4 mm. Each electrical stimulation event consisted of four minutes of data acquisition, including the first two minutes of baseline measurements. Relative change in cerebral blood volume (CBV) was estimated relative to the baseline. The thalamic electrical stimulation was applied at *t* = 61 s.

### 2.3. Estimation of microDoppler image

The acquired Doppler ensemble *E(x, z, t)* of ultrasound images were rearranged in to a spatiotemporal Casorati matrix *S*_*tissue*+*blood*_(*s*, *t*), where *s* and *t* denoted spatial and temporal coordinates, and the tissue clutter signal was suppressed using singular value decomposition (Demené *et al* 2015). A global singular order threshold was used for separating tissue clutter from blood flow signal. Subsequently, *S*_*blood*_(*s*, *t*) was transformed from two-dimensional Casorati form to three-dimensional Cartesian form ***E***_*blood*_(***x***, ***z***, ***t***). The clutter-filtered Doppler ensemble ***E***_***blood***_(***x***, ***z***, ***t***) were coherently integrated to estimate the microDoppler image MBF(*x*, *z*, *T*), where *T* denotes the acquisition time-stamp. For improved visualization of cerebral microvessels, the background noise bias in the microDoppler images were suppressed with respect to its clutter-filtered Doppler ensemble, as presented in Nayak *et al* (2019).

The functional activation maps (FAM) display changes in CBV at any time instance, relative to the baseline signal. The FAM images were estimated by subtracting time-averaged baseline signal *MBF*_*ref*_(*x*, *z*) from the individual *MBF*(*x*, *z*, *T*). In figures 2(d), (e), FAM image depicts the functional activation in the rat brain, overlaid on the *MBF*_*ref*_ image for improved interpretation and understanding. The corresponding correlation image and *z*-score images are depicted in figures 2(g), (h), respectively. Specifically, image (g) corresponds to correlation between the measured fUS signal at each pixel and the applied activation function depicted in blue in (f). Subsequently, the *z*-score image (h) was estimated corresponding to (g), with respect to the mean and standard deviation of the entire image. Both the correlation (−1 to 1) and *z*-score images display clear activation at the PMC and VLT regions, as also observed in the fUS image (e).

## 3. Results

### 3.1. fUS reveals time-locked brain activation evoked by thalamic DBS

DBS of VL thalamus evoked time-locked CBV changes in the rat brain (figure 2). Representative microDoppler image of the rat brain across the sagittal plane (ML = 1.4) displays a rich network of microvascular blood vessels of varying dimensions and orientations (figure 2(d)). Specifically, the cortical vessels were densely packed, and the location of the implanted electrode was visible due to the attenuation of the ultrasound signal. The observed changes in CBV were relative to the baseline blood flow measurements that were always recorded prior to application of the electrical stimulation at the thalamus (figure 2(e)). To ensure high imaging sensitivity in revealing CBV fluctuation, the fUS images were acquired through a craniotomical window, across the acoustically transparent dura-matter. Accordingly, the noise level in the base-line and stimulation phases of the fUS recording were considerably lower than the activation signal. The estimated CBV changes corresponding to the thalamic stimulation of the rat brain were overlaid on the microDoppler images, which highly co-registered with the anatomical features (figures 2(d), (e)). The increase in CBV was time-locked with VL thalamic DBS, and was observably specific to the PMC (figure 2(f)) that was repeatable and reproducible across the sequence of five consecutive pulses. However, even though the electrical stimulation was applied for 10 s (at *t* = 61 s), the corresponding CBV changes lasted observably longer than the stimulation period, which depended on the frequency and intensity of the stimulation. The correlation and *z*-score images (figures 2(g), (h)) further confirm that the activation observed in the rodent brain was synchronous with the activation function.

### 3.2. fUS detects the impact of electrical stimulation parameters on brain activation

**Figure 3.**
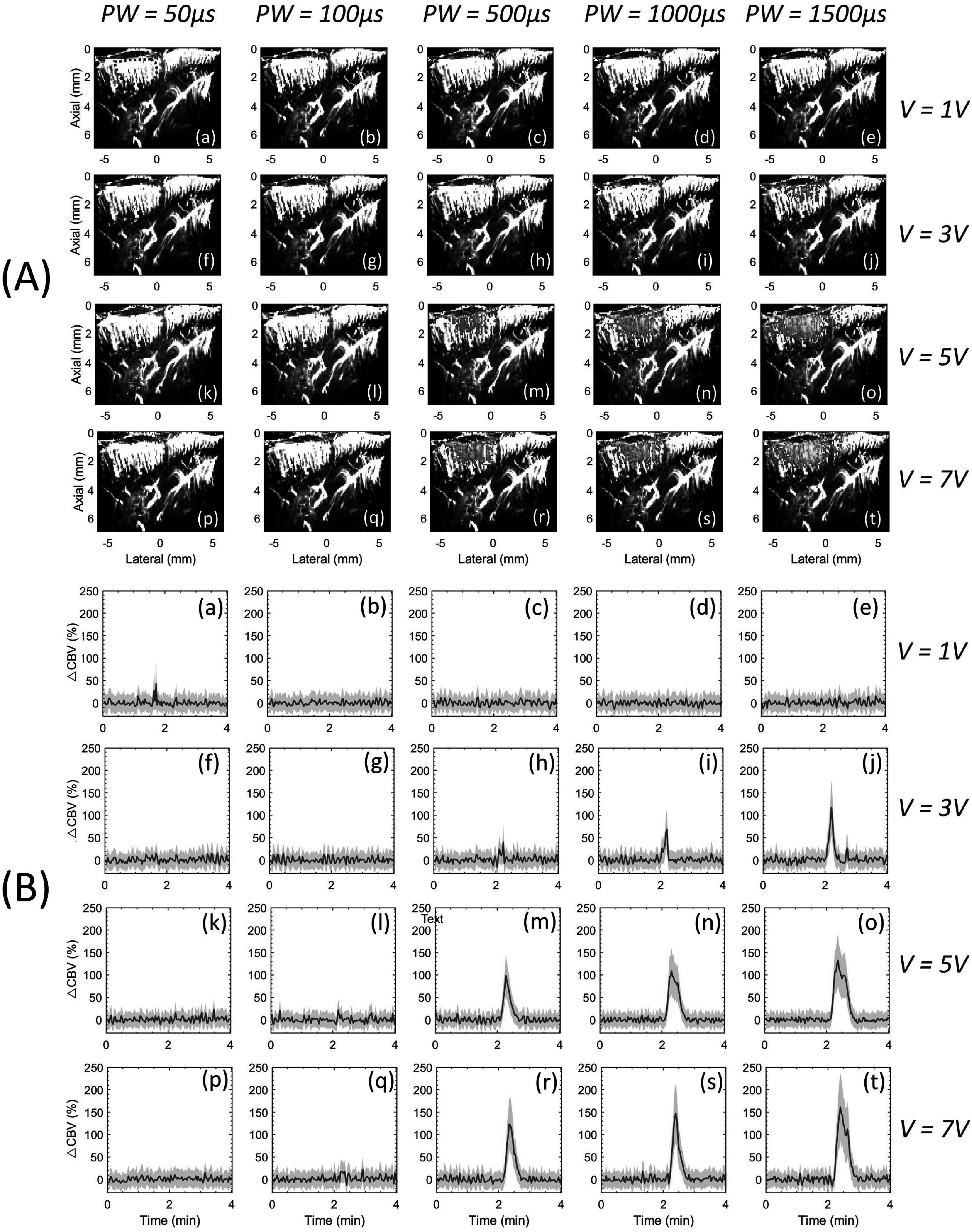
(A). Montage of FAM images displaying fUS activation corresponding to low frequency deep brain stimulation at the ventrolateral thalamus. Rows correspond to voltages 1, 3, 5 and 7 volts, whereas columns correspond to pulse width of 50, 100, 500, 1000, 1500 *μ*s. The colorbars are indicated in figures 2(d), (e). (B). Montage of temporal profile of fUS activation in the primary motor cortex, corresponding to low frequency stimulation at the ventrolateral thalamus. Rows correspond to voltages 1, 3, 5 and 7 volts, whereas columns correspond to pulse width of 50, 100, 500, 1000, 1500 *μ*s.

**Figure 4.**
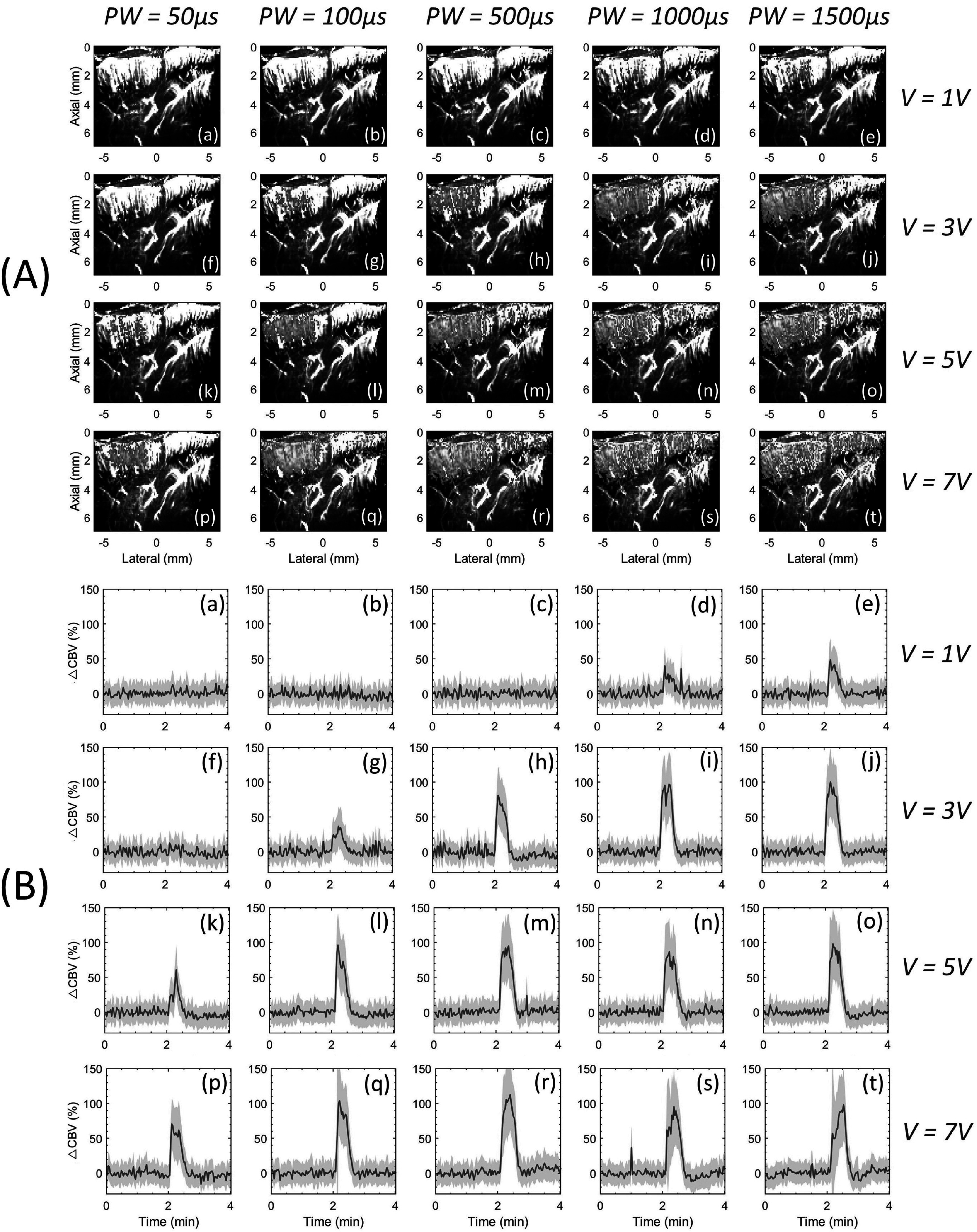
(A). Montage of FAM images displaying fUS activation corresponding to high frequency deep brain stimulation at the ventrolateral. Rows correspond to voltages 1, 3, 5 and 7 volts, whereas columns correspond to pulse width of 50, 100, 500, 1000, 1500 *μ*s. The colorbars are indicated in figures 2(d), (e). (B). Montage of temporal profile of fUS activation in the primary motor cortex, corresponding to low frequency stimulation at the ventrolateral thalamus. Rows correspond to voltages 1, 3, 5 and 7 volts, whereas columns correspond to pulse width of 50, 100, 500, 1000, 1500 *μ*s.

To visualize the impact of stimulation parameters on cerebral activation of the rodent brain, we performed DBS-fUS imaging with low (LFS) and high (HFS) frequency stimulations, across a range of signal intensities regulated by voltage and pulse width (PW). Figures 3(A) and 4(A) visualized site-specific CBV changes in the motor cortex of the rat brain, which corresponded with neural activation expected in the PMC in response to thalamic DBS. Figures 3(B) and 4(B) displayed the temporal characteristics of the CBV changes at the PMC, in response to 10s of thalamic DBS applied at *t* = 61 s.

**Figure 5.**
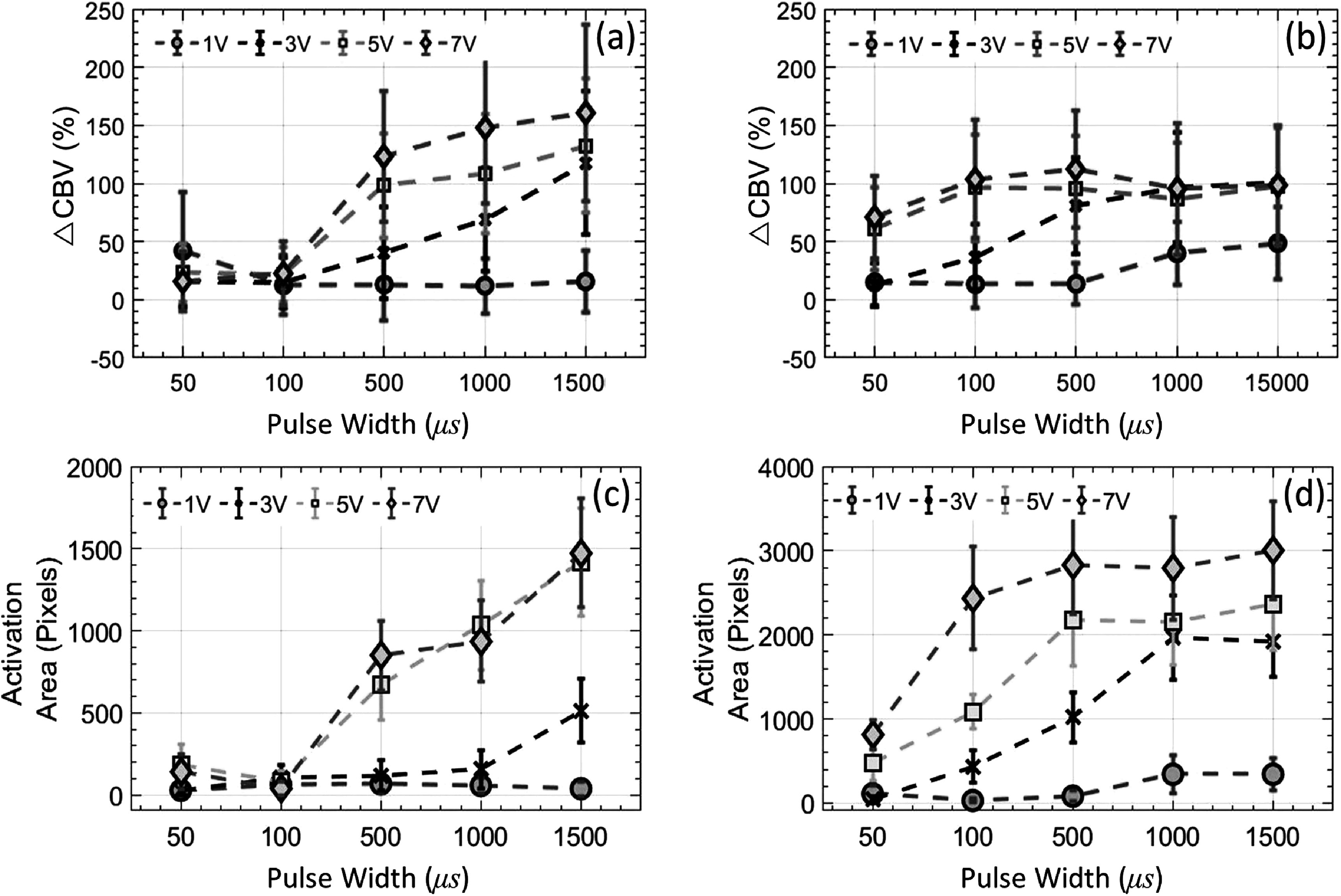
Quantitative plots displaying the variation in the cortical fUS signal with respect to low (left column) and high (right column) frequency deep brain stimulation at the ventrolateral thalamus. Top row quantifies the increase in fUS activation in the primary motor cortex as a function of voltage, pulse width and frequency of electrical stimuli. Bottom row quantifies the increase in area cortical activation area, corresponding to variation in voltage, pulse width and frequency of the electrical stimuli. The low and high frequency stimulation were applied at 10 and 100 Hz, respectively. The area of cortical activation was quantified with a threshold of 30 dB signal drop relative to the peak.

The LFS were highly site-specific to the activation of the PMC (figure 3), with increase in stimulation intensity corresponding to an reciprocal increase in the CBV amplitude. Specifically, no measurable change in CBV was observed at low voltage (1 V) or at low PW (50 and 100 *μ*s); at 3–7 V, only higher PW (500–1500 *μ*s) stimulations evoked cerebral activation, which consistently increased with V and PW (figure 5). For example, at PW = 1500 *μ*s, ΔCBV increased from 15.62% at 1 V, 117.4% at 3 V, 132.7% at 5 V to 161% at 7 V. Similarly, at fixed voltage of 7 V, average Δ CBV increased from 15.54% at 50 *μ*s, 123.1% at 500 *μ*s, 147.4% at 1000 *μ*s, to 161.1% at 1500 *μ*s. Further, the area of activation (AoA) increased with voltage and PW. However, the effect of PW was relatively more prominent than voltage, since no significant increase in AoA was observed beyond 5V. On the contrary, HFS led to consistent increase in AoA with voltage and PW. Particularly, the effect of voltage was relatively more predominant that PW, as the AoA associated PWs 500–1500 *μ*s were relatively similar at 5 and 7 V, respectively. Further, unlike in LFS, HFS were also particularly efficient in evoking CBV changes even at low voltage and PW (figure 4). A measurable increase in CBV was observed at low voltage (1 V) and at low PW (50 *μ*s). Specifically, at 1 V, a 40.1% and 48.6% increase in CBV was associated with PW of 1000 and 1500 *μ*s, respectively; at low PW (50 *μ*s), a 60.83% and 70.61% increase in CBV was associated with higher voltage of 5–7 V, respectively (figure 5). The increase in Δ CBV with voltage (3–7 V) and PW (100–1500 *μ*s) saturated at approximately 98% (figure 5). These results show that the LFS and HFS at VL thalamus evoked substantial neural response, which varied with stimulation intensity. Overall, at low signal intensity, HFS was more efficacious in evoking neural response. Whereas, the highest increase in Δ CBV was associated with LFS, specifically at high signal intensity, which was 63% higher than that observed with HFS (figure 5).

## 4. Discussion

This study reports the feasibility of using fUS imaging to characterize cerebral hemodynamic changes in rat brain associated with DBS. The high imaging frame-rate and spatial resolution of fUS imaging enabled us to visualize time-locked and site-specific changes in CBV associated with DBS-evoked brain activity that were sensitive to the electrical stimulation parameters.

Motor thalamus is a well-established DBS target for essential tremor since it plays an important role in relaying functional information to the cerebral cortex related to external stimuli (Pollak *et al* 1993, Pahwa *et al* 2001, Schuurman *et al* 2008). The fUS imaging successfully visualized VL thalamic DBS-induced increase of CFB at the PMC (figures 2, 3(A) and 4(A)), and this modulation of CBV was time-locked with the electrical stimulation (figures 2, 3(B) and 4(B)). This observation was consistent with the presence and activation of the well corticothalamic anatomical connections in the rodent brain (Raethjen *et al* 2007, Raethjen and Deuschl 2012). The communication between the cerebral cortex and the thalamus during sensory processing is achieved by sensory transmission and reception between the thalamic and corticothalamic feedback neurons (Briggs and Usrey 2008). In a recent study in rodents, we used electrophysiology to demonstrate that DBS at the VL thalamus, which is a homolog for VIM in humans, significantly affects neuronal firing patterns at the PMC (Lee and Chang 2019). In this paper, CBV changes quantified using fUS imaging demonstrated that thalamic DBS led to activation of the cerebral cortex, specifically at the PMC (figure 2). Our current preliminary data suggests that fUS imaging as an investigational tool to study stimulation-associated CBV changes corresponding to connected functional circuits. The DBS-evoked CBV changes visualized using fUS signal was time-locked with electrical stimulation of the VL thalamus. This demonstrated that the CBV changes quantified using fUS imaging were indeed triggered in response to thalamic DBS, which were repeatable and reproducible, both temporally and spatially (figure 2).

The therapeutic outcome of DBS is known to be dependent on stimulation parameters and the target. Studies have shown that HFS ranging from 60 to 1000 Hz play an important role in symptom relief. However, thalamic LFS have demonstrated aggravation of tremor activity (Bejjani *et al* 2000, Kuncel *et al* 2007). Further, it has also been observed that LFS leads to myoclonus in non-tremor patients, indicating the presence of a central oscillatory mechanism that involves the thalamus and the olivocerebellar complex (Constantoyannis *et al* 2004). LF-DBS stimulation of the subthalamic nucleus has also demonstrated LFS-dependent symptom aggravation (Moro *et al* 2002, Kuncel *et al* 2007, Lai *et al* 2014). Cellular response based computational modeling of extracellular thalamic stimulation demonstrated reduced output firing rate of thalamo-cortical neurons upon applying HFS, whereas LFS increased the amplitude of intrinsic burst activity and thus enhanced the output firing rate (Kuncel *et al* 2007). In previous studies, high frequency thalamic DBS (150–10 000 Hz) suppressed tremor at low current amplitude (Benabid *et al* 1991); however, at low frequencies, increased voltage was necessary to produce the similar effects (Benabid *et al* 1991, Limousin *et al* 1995, Kuncel and Grill 2004), which was thematically consistent with our observations reported in this study (figure 5). Further, in our previous study, we found that HFS reduced the BOLD signal during the short-term stimulation (Paek *et al* 2015). Here, we also observed that HFS-induced activation was significantly lower than LFS-induced activation at the PMC (figure 5).

It is possible that some of the activation might be caused by thermal effects. Since we did not directly measure the temperature at the stimulation site, we cannot completely rule out this possibility. However, considering that the activation area was still highly focused inside the PMC and the local area of the stimulation electrode tips was not overly activated, the temperature effect could be minimal. If the temperature rise around the electrode tips affects the blood flow, the local area around the electrode tip would be the most affected area and the activation would be distributed evenly around the electrode. In addition, even with the highest voltage, the activation was stably generated with the repetitive stimulation. This suggests that the stimulation did not damage the tissue around the electrode. If the temperature rise might happen, the degree would be minimum. In our experiment, we carefully checked the condition of animals and confirmed they are alive under anesthesia. In live animals, the circulation system works normally. Thus, in the normal healthy circulation condition, the minimally raised temperature will be easily diffused. Finally, the center of the area activated by high voltage stimulation originated from the same spot as that by low voltage stimulation. For these reasons described above, we believe that the temperature effect will be minor.

An important question in the context of this study is how closely does the fUS signal compare with BOLD signal estimated using fMRI, the current gold standard in functional neuroimaging. fUS is characterized by changes in CBV (figure 2), which is estimated using ultrafast ultrasound Doppler imaging in high spatial and temporal resolution (Macé *et al* 2011, Mace *et al* 2013, Tanter and Fink 2014, Deffieux *et al* 2018). Recent investigations using two-photon microscopy have reported that fUS robustly represents neuronal activation across a wide range of stimulation paradigms and pre-clinical models (Aydin *et al* 2020). In a co-registered single voxel brain volume, transfer functions were determined to characterize the relationship between dendritic calcium signals of specific neurons and vascular signals measured at mesoscopic levels. Further, measurement of site-specific neuronal activity based on estimation of CBV is potentially more sensitive than the BOLD signal (Miller *et al* 2001, Aguirre *et al* 2002, Tjandra *et al* 2005, Liu and Brown 2007, Osmanski *et al* 2014). Specifically, in the BOLD signal model, neural activation-induced fractional BOLD signal changes are related to combined underlying changes in the CBV and CMRO_2_ (Davis *et al* 1998, Hoge *et al* 1999, Liu 2013). Accordingly, by directly measuring the changes in the microvascular hemodynamics, fUS measurements are less impacted by the confounding factor of blood CO_2_ and CMRO_2_ concentration, which can change with the respiration rate, thus incurring changes in BOLD signal unrelated to neural activity (Osmanski *et al* 2014). Further, this is consistent with flow-sensitive MRI imaging studies that revealed CBV variations were coherent with spatial patterns seen with BOLD signal in response to resting state and task-enabled activation (Vern *et al* 1988, Golanov *et al* 1994, Biswal *et al* 1997, Fukunaga *et al* 2008, Wu *et al* 2009). However, even though estimation of CBV is expected to be biologically more specific to correlate with neural activity, MRI based measurements of CBV have significantly lower sensitivity and temporal resolution than BOLD imaging (Liu and Brown 2007). fUS imaging directly addresses this trade-off between specificity and sensitivity associated with MRI based neuroimaging by measuring CBV changes in high sensitivity and spatial and temporal resolution, applying concepts of ultrasound Doppler imaging.

This study has three main limitations. First, the experiments on anesthetized rats enabled us to demonstrate the feasibility of using fUS imaging for measuring DBS-evoked neurological responses. Advancing this fUS study to awake and fully behaving animals may show a relative increase in overall cerebral response over anesthetized animals, as anesthesia may impact neurological metabolism and cerebrovascular response resulting in reduced cerebral activation (Chao *et al* 2014). Second, the pilot study has low statistical power since it was performed on a limited population of rats. Third, fUS imaging was performed using a linear-array probe that scanned a single cross-section of the rat brain. The chosen imaging cross-section depicted the maximum extent of the region-of-interest (i.e. PMC). Further, as previously demonstrated (Rabut *et al* 2019), fUS technology can be extended for high frame-rate 3D volume imaging using matrix arrays connected to ultrasound machines with 1024 input-output channels. As the next step, we will advance 3D fUS imaging technology for neuroimaging of DBS-evoked cerebral response in awake and behaving animals (Urban *et al* 2015, Tiran *et al* 2017).

## 5. Conclusion

This is the first study that demonstrates the feasibility of using functional ultrasound imaging for characterizing brain activity in response to DBS. The thalamic stimulation led to activation in the PMC, which varied significantly in response to frequency of the stimulation. The encouraging preliminary results paves the way for developing functional ultrasound imaging for visualizing neurological activity in awake and behaving rats in response to DBS of different targets for motor-related disorders.

## Disclosure of conflict of interest

The authors have no financial interest to disclose related to the content of this article.
